# Research patterns in sports law and sports governance: a scopus bibliometric study

**DOI:** 10.3389/fspor.2025.1590858

**Published:** 2025-07-15

**Authors:** Marina Kamenecka-Usova, Zanete Korde, Janis Zidens, Signe Luika, Olena Agapova, Karina Palkova, Kristine Martinsone

**Affiliations:** ^1^ Faculty of Social Sciences, Rīga Stradiņš University, Riga, Latvia; ^2^ Department of Health Psychology and Pedagogy, Rīga Stradinš University, Riga, Latvia; ^3^ RSU Latvian Academy of Sport Education, Rīga Stradinš University, Riga, Latvia

**Keywords:** sports law, sports governance, lex sportiva, good governance, bibliometric analysis

## Abstract

This bibliometric study explores research patterns in lex sportiva, sports law and sports governance, analyzing data retrieved from Scopus between 1977 and 2024. The study examines publication trends, citation networks, keyword co-occurrence and the most influential sources to identify key themes, leading authors and reputable journals. The results indicate that while both fields have been extensively studied, their intersection remains relatively underexplored, with “good governance” emerging as the primary link between sports law, lex sportiva, and governance studies. The citation analysis highlights regional disparities, with Australia, the United States, and the United Kingdom leading in research impact, while countries such as China and Germany exhibit high output but lower citation influence. Co-authorship networks reveal strong collaborations within Europe and North America but limited engagement from other regions. The findings underscore the growing importance of sports governance and legal frameworks in addressing contemporary challenges, particularly within the European Sport Model. This study provides valuable insights for researchers and policymakers, emphasizing the need for interdisciplinary approaches to further advance the field.

## 1 Introduction

Sports law and sports governance are increasingly recognized as pivotal domains in the regulation and oversight of global sport. However, as the authors suggest, these fields have traditionally evolved in parallel, with limited integrative analysis. This study addresses this gap by pursuing the objective of systematically analyzing the evolution, thematic structure, and scholarly interlinkages between sports law (with an emphasis on *lex sportiva*) and sports governance through bibliometric methods. The aim is to uncover patterns of publication, collaboration, and conceptual convergence that characterize the academic discourse at the intersection of these two fields.

Research on sports law and sports governance is crucial for enhancing the effectiveness and integrity of the sports sector. Sports law establishes the foundational legal frameworks for regulating organizations, while governance focuses on the systems and processes that ensure operational success and accountability.

Together, they enable effective risk management, align organizational policies with legal standards, and ensure ethical behavior across the sector.

This collaboration is particularly relevant in the context of the European Sport Model (1), which emphasizes solidarity, openness, and a hierarchical structure connecting professional and grassroots levels. The model's principles are supported by EU initiatives, such as Article 165 of the Treaty on the Functioning of the European Union (TFEU), which highlight transparency, fairness, and inclusivity in governance practices. These efforts are reinforced by lex sportiva, the legal framework established by international sports organizations, which operates alongside European Union (furthermore, EU) oversight to ensure harmonized governance across borders. A key element within this framework is the concept of the autonomy of sport, which refers to the right of non-governmental, non-profit sports organizations to regulate their internal affairs independently, within the scope of national, European, and international law. This includes the freedom to establish, amend, and interpret sport-specific rules without undue political or economic influence; to democratically elect their leadership without interference; to receive and utilize funding with proportionate obligations; and to develop standards in consultation with public authorities that are legitimate and aligned with their objectives. This principle of conditional autonomy is essential to maintaining the integrity and self-regulation of the sporting sector (2).

Institutions like the Union of European Football Associations (furthermore, UEFA) and the Court of Arbitration for Sport (furthermore, CAS) play key roles in advancing these standards, with CAS facilitating the resolution of disputes and setting precedents for global sports law. The EU's involvement further illustrates the importance of aligning governance with legal frameworks to address challenges like commercialization, regulatory inconsistencies, and governance failures.

In this regard, a bibliometric analysis based on Scopus offers a robust method to study the intersection of sports law and governance. By leveraging bibliometric tools, stakeholders can gain valuable insights into how these intertwined domains are being studied globally, providing a data-driven foundation for shaping future research and policy initiatives.

Before proceeding, a brief *introduction to the theoretical foundations and key concepts*—including sports law, *lex sportiva*, and sports governance—is provided, as these are essential for contextualizing and informing the subsequent analysis.

In 1977, Bowie K. Kuhn, expresses a critical view of the term “sports law.” Kuhn argues that “sports law” is a misleading term, as it suggests the existence of a distinct, independent legal field. He believes this obscures the reality that professional sports operate under the same legal principles and frameworks as other industries. He contends that the term oversimplifies the complex relationship between sports and the law. This relationship involves intricate interactions between psychological, social, and economic forces. Kuhn attributes the popularity of the term to the significant public interest and emotionalism surrounding professional sports. He highlights how this atmosphere often leads to oversimplification, misunderstanding of precedents, and the perpetuation of myths. He also references the myth that the Supreme Court has declared baseball is not a business but a sport clarifying that this is incorrect and that the Court has explicitly characterized baseball as both a sport and a business.

Overall, Kuhn sees the term “sports law” as doing a disservice to the industry by masking the complexities and multidisciplinary nature of the legal issues involved in professional sports (3).

An opposing view is expressed by John Weistart and Cym Lowell in 1979. Authors observed that based upon their research it became clear that there were many areas in which sports-related problems required a specially focused analysis. On some matters, there are legal doctrines which apply in the sports area and nowhere else. This is the case, for instance, with respect to such diverse matters as baseball's antitrust exemption and some of the tax rules to be applied to the recapture of depreciation on player contracts (4). Weistart and Lowell conclude their analysis by emphasising areas in which the factual uniqueness of sports problems require specialised analysis. In this regard, they caution courts to take care in drawing analogies. Thus, while not expressly adopting the position that recognises the existence of a course of study called “sports law”, Weistart and Lowell strongly suggest that two phenomena, the unique application of legal doctrine to the sports context and the factual uniqueness of sports problems that require the need for specialised analysis, support the notion that a body of law called “sports law” might exist (4).

Comparing the two views on “sports law” reveals distinct perspectives:

*Focus on Uniqueness:* Kuhn downplays the need for a distinct legal field for sports, emphasizing general legal principles, whereas Weistart and Lowell highlight the factual and doctrinal uniqueness of sports problems, supporting the idea of specialized analysis.

*Terminology:* Kuhn rejects the term “sports law” as misleading, while Weistart and Lowell cautiously suggest that the unique application of legal doctrines in sports could justify such a term.

*Approach to Legal Issues:* Kuhn emphasizes broad applicability of existing legal frameworks, while Weistart and Lowell argue for courts to exercise care in applying analogies due to the unique nature of sports-related issues.

Overall, Kuhn's perspective is critical and generalized, whereas Weistart and Lowell take a more nuanced view, recognizing unique legal challenges in sports that might warrant specialized analysis.

In 2001, Timothy Davis wrote a paper exploring what is sports law, explaining three main views on this issue:

1) no separately identifiable body of law exists that can be designated as sports law and the possibility that such a corpus of law will ever develop is extremely remote;
2) although sports law does not presently represent a separately identifiable substantive area of law, recent developments suggest that in the near future it will warrant such recognition; or
3) a body of law presently exists that can appropriately be designated as sports law (5).

Hence, traditionalists, such as Bowie K. Kuhn, argue that sports law is not a unique field but merely an application of general legal principles in the sports context. Moderates, such as Kenneth Shropshire (6) and Burlette Carter (7), acknowledge that sports-specific legal issues are emerging but believe the field has not yet matured into a distinct corpus of law. Modernists, led by Simon Gardiner (8), assert that sports law has become a substantive area due to growing legislation, case law, and its increasing economic importance, despite past intellectual marginalization. Gardiner concludes, “As an area of academic study and extensive practitioner involvement, the time is right to accept that a new legal area has been born—sports law.”

Building on the debate surrounding the nature and scope of sports law, it is essential to explore the related concept of lex sportiva, which expands the discussion into the global and transnational dimensions of sports regulation. While sports law is often rooted in national legal frameworks or informed by broader governance principles, lex sportiva emerges as a distinct legal system, shaped by international sports organizations and designed to govern the unique and complex relationships within the global sports community. This shift from national legal frameworks to an autonomous, international sports legal order highlights the evolving nature of legal regulation in the sporting world.

In this regard, it is important to highlight the series of scholarly works on the formation and concept of lex sportiva by a European academic, hailing from the region where the Olympic Games originated. According to Dimitrios P. Panagiotopoulos, lex sportiva, or “global sports law,” is a unique and independent legal system developed by international sports organizations, such as the International Olympic Committee (IOC) and International Sports Federations (ISF). It exists outside the traditional state-based legal framework and operates as a sui generis international legal order. It governs sports-related relationships and activities, shaping a private, non-domestic law specific to the global sports community.

The pyramid of governance might be discribed as follows:

1) At the top of the sports law hierarchy is the IOC, which legitimizes and recognizes the ISFs and National Olympic Committees under the Olympic Charter.
2) ISFs, private entities governed by the laws of their respective seats, regulate their respective sports across national boundaries.
3) National federations are required to harmonize their regulations with the rules of the ISFs, often incorporating lex sportiva into their national legal systems to ensure compliance.

Hence, the characteristics are following:

*Autonomy*: Lex sportiva operates parallel to and independent from traditional state legal systems, regulating international sports activities in a “borderless” environment.

*Binding Nature:* Although established by private entities, its rules are binding on athletes, federations, and other stakeholders for practical and necessity reasons.

*Conflict with National Law:* Its imposition often creates tensions with domestic legal systems and supranational laws, such as those of the European Union, especially in matters of individual freedoms and financial regulations, e.g., Bosman case (9), Walrave and Koch (10). Lex sportiva, as stated by Panagiotopoulos shares characteristics with lex mercatoria, the private international law governing global commerce. Both systems are created by non-state entities, function internationally, and have limited state intervention.

Lex sportiva encompasses rules addressing doping disputes, athlete-vs.-federation conflicts, and CAS decisions. These areas highlight the practical application of lex sportiva as a “global sports law,” emphasizing its role in managing international sports and Olympic relations. Lex sportiva combines elements of international law (its global scope and regulation) and domestic law (enforcement mechanisms and incorporation into national legal systems). It forms a new kind of legal order, imposing heteronomous rules on the global sports community.

In conclusion, Panagiotopoulos views lex sportiva as a distinct and evolving legal framework that governs global sports relationships through principles set by international sports institutions. While it exists independently from traditional state law, it often interacts with and challenges national and supranational legal systems.

In turn, the term “sports governance” is relatively well-defined in the academic literature, and its conceptual boundaries are largely uncontested. In a recent and topical article, Cho, Conrad, Holden, and Dodds propose a definition that, according to the authors, encompasses most of the essential components emphasized in previous studies. They define sports governance as “the exercise of granted power and authority to monitor, direct, manage, and control a sport organization's strategic performance and compliance with relevant regulations and laws, taking into account internal dynamics and the external environment” (11). Most frequently, the concept of sports governance is examined through the lens of normative standards and best practices, collectively referred to as good governance. This notion will be analyzed in greater detail in the subsequent chapters of this study.

The interplay between lex sportiva and sports governance becomes apparent when considering how the principles and rules established by international sports institutions are implemented within governance frameworks. While lex sportiva provides a legal foundation for regulating global sports relationships, sports governance translates these principles into practical systems and processes that ensure transparency, accountability, and ethical management. This connection is particularly evident in the European Union, which serves as a model for integrating good governance principles into sports administration through a combination of soft law and best practices. Such governance efforts are essential for maintaining public trust and safeguarding the integrity of sports organizations.

The remainder of this paper is structured as follows. Chapter 1 describes the methodological framework, including data collection procedures and bibliometric tools employed. Chapter 2 presents the results of the bibliometric analysis, including publication trends, citation metrics, authorship patterns, and thematic clusters. Chapter 3 provides a critical discussion of these findings, highlighting conceptual overlaps, especially a bridging concept between sports law and governance. Last chapter concludes the paper with a summary of key insights, implications for future research, and recommendations for scholarly integration across legal and governance domains in sport.

## 2 Methodology

According to Mukherjee et al., bibliometric research is a variant of systematic literature review that applies quantitative and statistical techniques—such as descriptive statistics, performance analysis, and science mapping—to bibliographic data like publications and citations. Unlike PRISMA-guided systematic reviews, which are designed to answer specific research questions through a rigorous, often qualitative process of study identification, screening, and critical appraisal based on predefined criteria (12), bibliometric analysis leverages advanced technologies, including big data analytics and machine learning, to objectively and comprehensively analyze large volumes of literature (13). This quantitative approach enables the mapping of publication trends, co-authorship patterns, and thematic structures, as well as the visualization of citation and keyword networks. Bibliometric methods minimize subjectivity by relying on data-driven techniques, allowing for the evaluation of productivity and impact, the identification of knowledge clusters and relationships within a field, and the analysis of hundreds to thousands of articles. As a result, bibliometrics provides extensive insights into a domain, including identifying major contributors (authors and institutions), mapping relationships among publications and topics, and highlighting research gaps, hidden biases, and underrepresented populations. Additionally, bibliometric research often employs advanced tools and techniques, including software like CiteSpace and VOSviewer, as well as databases such as Scopus and Web of Science, to generate both visual and non-visual data representations. Furthermore, it aims to advance theory by moving beyond descriptive analysis, linking findings to theoretical contributions, and identifying directions for future research (14). In line with the capabilities of bibliometric research, Scopus emerges as an ideal choice for this study due to its comprehensive coverage of peer-reviewed literature across a wide array of disciplines. According to the academic publisher Elsevier, Scopus provides access to an extensive database of abstracts and citations, enabling efficient discovery of authoritative research, identification of leading experts, and generation of strategic insights through its robust metrics and analytical tools (15). Compared to other databases, Scopus offers robust citation data and advanced analytical tools, facilitating detailed mapping of publication and citation trends. However, authors acknowledge that relying solely on Scopus may exclude relevant publications indexed exclusively in other databases such as Web of Science or regional repositories. This limitation may affect the representation of certain regions or non-English language scholarship. Future studies should consider a multi-database approach to provide a more exhaustive overview. The subsequent bibliometric analysis was conducted using Biblioshiny (part of the “Bibliometrix” package in R, version 4.1) and VOSviewer (version 1.6.20) to visualize the aggregated metadata from the cataloged articles. Table 1 outlines the step-by-step process of data collection, curation, analysis, and visualization employed in the bibliometric analysis.

**Table 1:** Phases of the bibliometric analysis: data collection, curation, and visualization.

Nr.	Research phase	Output	Software/tools used
1	Establishing the research parameters	The scope of the bibliometric analysis was determined	Authors's contribution
2	Data source identification	A suitable database was selected to ensure extensive coverage of high-quality literature. Searching was performed within categories: article title, abstract, keywords. A total of 442 papers related to the topic were initially retrieved.	Scopus
3	Data curation	The dataset underwent filtering based on specific inclusion and exclusion criteria, such as limiting results to articles, reviews, and English-language publications yielding a final dataset of 308 articles for detailed analysis.	Manual screening complemented automated searches to ensure appropriateness
4	Data extraction	Relevant metadata, including titles, abstracts, keywords, citations, and author information, was exported from Scopus for further examination.	CSV file containing bibliometric metadata
5	Keyword and author consolidation	To ensure consistency and reduce redundancy, synonyms and variations of key terms were consolidated. Manual evaluation played a critical role in grouping similar keywords, enabling a more accurate representation of broader research trends.	Thesaurus file
6	Performance evaluation	Key performance metrics were analyzed, including annual publication trends, leading corresponding authors, and citation distributions by country.	Biblioshiny
7	Connection strength assessment (science mapping)	Link strength analysis was performed to uncover keyword co-occurrence, collaboration across countries and collaborative patterns examined through the citation analysis of key contributors.	VOSviewer
8	Visualization	The bibliometric results were visualized, with outputs emphasizing dominant clusters and highlighting key entities within the research landscape.	VOSviewer, Biblioshiny
9	Interpretation and reporting	A comprehensive discussion was presented, synthesizing findings and emerging trends while incorporating insights from network visualization and link strength analysis.	Author's subjective evaluation

The research phases that need additional clarification will be described in greater detail below.

### 2.1 Data source selection and curation

The data for this study was retrieved from Scopus, a comprehensive abstract and citation database widely used for bibliometric analyses. The initial search phrase “sport governance” AND “lex sportiva” returned no results, suggesting that “lex sportiva” and “sports governance” are not frequently studied together in the existing literature or are not commonly linked in Scopus-indexed documents. To broaden the scope, the term “lex sportiva” was replaced with “sport law,” and the search phrase was modified to “sport governance” AND “sport law.” However, this search yielded only five results, which included studies addressing topics such as the European Superleague (16), EU competition law in sports governance (17), and the governance of Chinese professional football (18). While these articles were relevant, the limited number of results made the dataset insufficient for robust analysis.

To address this limitation, the search parameters were expanded using the phrase “sport governance” OR “lex sportiva,” which significantly increased the results to a total of 442 papers related to the topic were initially retrieved.

After data curation, which included filtering for articles published in English and categorized as either articles or reviews, a total of 308 documents were retrieved.

This included 291 articles and 17 reviews, indicating that there is substantial literature on each topic individually. This result suggests that “lex sportiva” and “sport governance” are relevant in their own right but may represent distinct or loosely connected areas of research.

This iterative approach to search term refinement ensured that the dataset adequately represented the broader research landscape of sports governance and its intersections with legal frameworks. It also highlights a potential gap in the literature, suggesting an opportunity for future studies to explore the linkages between these two domains.

### 2.2 Keyword consolidation

To streamline the dataset and enhance the clarity of bibliometric visualizations, keywords representing identical or similar concepts were unified using the thesaurus file. This process ensured consistent terminology and reduced redundancy, thereby improving the interpretability of bibliometric analysis.

Consolidation Process

1. Identifying synonyms and variants: Keywords that express similar concepts or are commonly used interchangeably were identified for consolidation.
2. Standardizing terminology: Singular and plural forms were standardized to avoid fragmentation.
3. Harmonizing minor linguistic difference: Terms reflecting minor linguistic differences were harmonized (Table 2).

**Table 2:** Examples of consolidated keywords.

Original keywords	Consolidated keywords
Boards; board	Board
Sport governance; sports governance	Sport governance
Sports for all; sport for all	Sport for all
Mega-sporting events; sport mega-events	Sport mega-events
Lex sportiva; lex sportive	Lex sportive
Wada; world anti-dopong agency; world anti-Dopong agency (wada)	World anti-dopong agency (wada)
National sport organisations; national sporting Organisations; national sports organizations	National sports organizations
Ioc; international olympic committee	International olympic committee

The same principle was applied to consolidate the authors of the available researches, e.g.,

“gonçalves g.h.t.” was consolidated under “goncalves g.h.t.”, “garmamo m.g.; haddera t.a.; tola z.b.; jaleta m.e.” was consolidated under “garmamo m.; haddera t.; tola z.; jaleta m.”

Consolidation process allowed to achieve the following:

Minimize visual clutter: By reducing the number of distinct nodes in bibliometric maps, dominant trends and patterns become more apparent.

Improve analysis: Unified keywords ensure that fragmented studies are cohesively analyzed, accurately reflecting research activity.

Enhance terminological consistency: The consolidation eliminates discrepancies caused by variations in spelling, formatting, or linguistic usage.

## 3 Results

### 3.1 Annual scientific production of research

**Figure 1:**
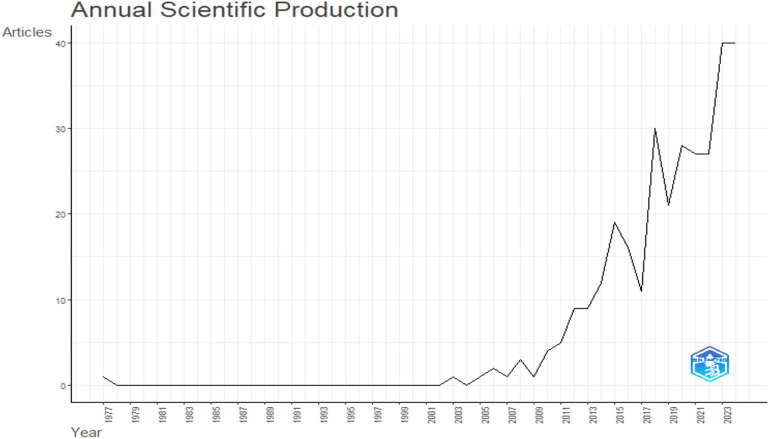
Annual scientific production. The graph displays the annual number of publications from 1997 to 2024.

Figure 1 illustrates the annual scientific output in lex sportiva and sports governance from 1977 to 2024. The graph begins with a single publication in 1977, followed by a significant hiatus with no publications from 1978 to 2002. This gap may reflect limited academic interest, the nascent stage of these research domains, or the lack of established research frameworks during that period.

The early 2000s marked the beginning of a gradual increase in publications, which accelerated notably from 2010 onwards. This growth aligns with several historical and societal developments that likely influenced academic interest. The globalization of sports, alongside the increasing prominence of international sporting events such as the FIFA World Cup and the Olympic Games, heightened public and academic interest in the governance and legal aspects of sports. The 1995 Bosman ruling in the European Court of Justice, for example, fundamentally reshaped the legal and economic landscape of European football, drawing attention to issues of lex sportiva and sports governance.

The acceleration in publication volume post-2010 can also be attributed to advancements in research methodologies, access to digital databases, and tools for bibliometric analysis, which enabled more extensive and interdisciplinary studies. Furthermore, changes in global sports policies, such as reforms by organizations like FIFA and the International Olympic Committee (IOC), as well as high-profile scandals like the 2015 FIFA corruption case (19), likely spurred academic interest in governance and legal frameworks.

From 2017 onwards, the trend indicates a particularly rapid increase in scientific output. This growth may have been driven by rising public and institutional awareness of governance challenges in sports, as well as legal controversies involving doping, match-fixing, and gender discrimination. Additionally, the integration of sports governance research with disciplines such as law, sociology, and economics expanded the scope of study, attracting a broader range of scholars.

By 2023, the field reached its peak with 40 publications, maintaining its high output into 2024. This sustained growth underscores the increasing recognition of lex sportiva and sports governance as vital areas of research, reflecting the ongoing evolution of the global sports industry and its legal and organizational complexities.

### 3.2 Corresponding authors by country

**Figure 2:**
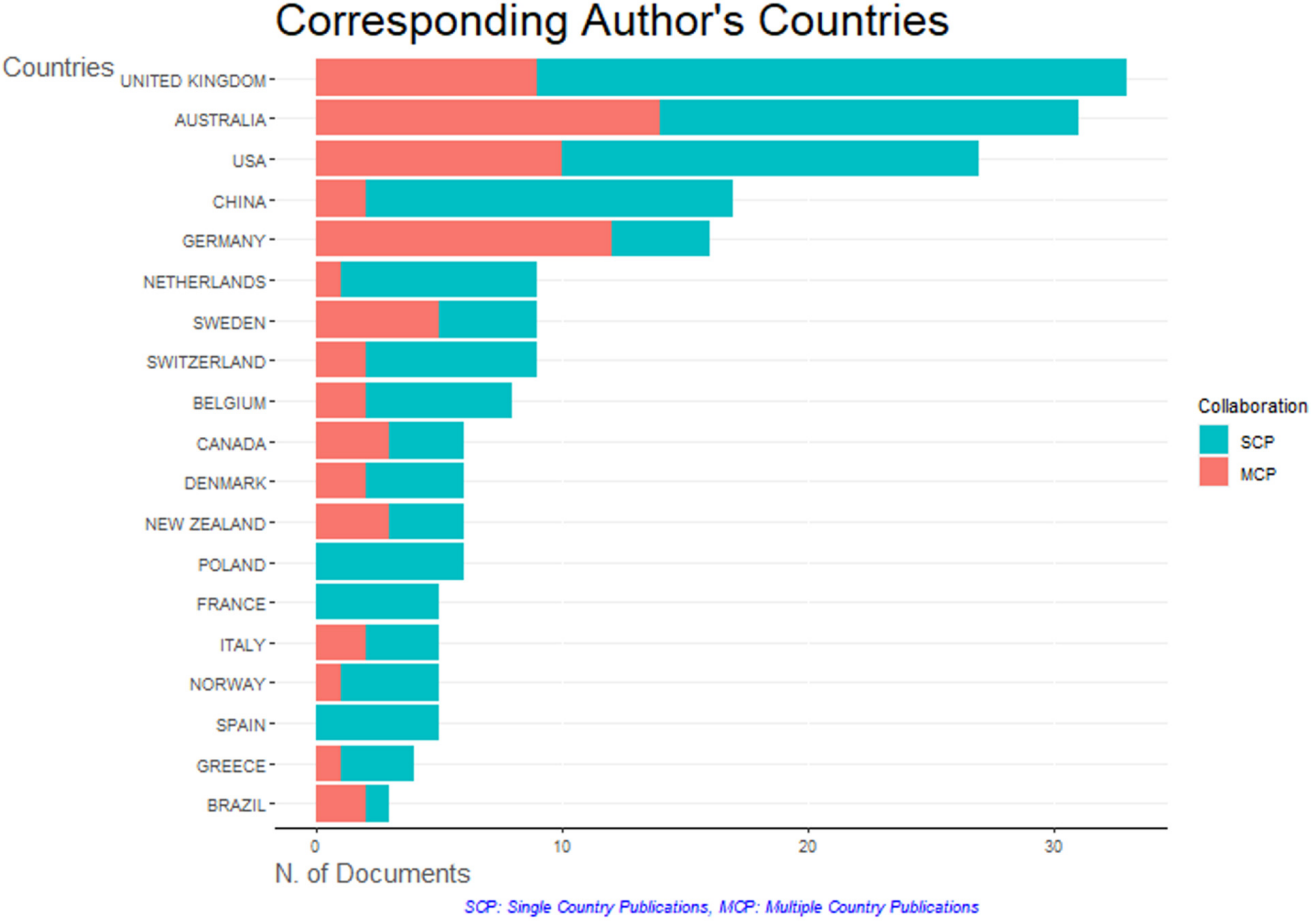
Corresponding authors. Graph show divers landscape of scientific collaborations across countries.

The analysis of corresponding authors reveals a diverse landscape of scientific collaboration across countries (Figure 2 and Table 3). The United Kingdom stands out as a significant contributor, with 33 articles, including 24 single-country publications (SCPs) and 9 multiple-country publications (MCPs), reflecting a balanced mix of domestic and international collaborations. This is evident in its moderate international engagement ratio (MCP_Ratio = 0.2727), i.e., proportion of articles in which there is at least one author with an affiliation in a country other than that of the corresponding author (20). Australia also contributed substantially with 31 articles, but with a higher emphasis on international collaboration (MCP_Ratio = 0.4516), indicating a strong inclination towards global partnerships. The United States, with 27 articles, demonstrates a strong focus on international collaboration as well, with an MCP_Ratio of 0.3704. Germany is notable for its high level of international collaboration, despite contributing fewer articles (16), with an MCP_Ratio of 0.75. This suggests significant involvement in international projects. Sweden also exhibits a strong international presence with an MCP_Ratio of 0.5556. In contrast, countries like Poland and France have an MCP_Ratio of 0, indicating that all their contributions are single-country publications. China and the Netherlands also have low MCP ratios (0.1176 and 0.1111, respectively), emphasizing more domestic contributions. Interestingly, smaller contributors like Brazil show a strong inclination toward international partnerships, with an MCP_Ratio of 0.6667 despite contributing only three articles. Overall, countries with fewer total articles often exhibit either very high or very low international collaboration, reflecting diverse research ecosystems. Major contributors like the UK, Australia, and the USA maintain a balance between domestic and international research output.The dominance of non-EU countries such as the UK, Australia, and the USA in this data can be attributed to several factors. English being the lingua franca of scientific research provides these countries with a natural advantage in publishing and participating in international collaborations without language barriers. Additionally, the EU's internal focus, such as programs like Horizon Europe, may lead to more collaborations within the EU, resulting in a higher number of single-country publications and less global visibility. Linguistic and cultural diversity within the EU can also create subtle barriers to collaboration compared to the more cohesive research ecosystems in non-EU countries. Historical and geopolitical factors also play a role, with non-EU countries like the USA and the UK having historically dominated academic publishing and global research networks, maintaining a legacy of influence. Australia's geographical position fosters collaborations with rapidly growing research economies in the Asia-Pacific region, further enhancing its global research presence.

**Table 3:** Corresponding authors. Table show diverse landscape of scientific collaborations across countries.

Country	Articles	SCP	MCP	MCP_ratio	MCP_ratio (%)
United Kingdom	33	24	9	0,27272727	27
Australia	31	17	14	0,4516129	45
USA	27	17	10	0,37037037	37
China	17	15	2	0,11764706	12
Germany	16	4	12	0,75	75
Netherlands	9	8	1	0,11111111	11
Sweden	9	4	5	0,55555556	56
Switzerland	9	7	2	0,22222222	22
Belgium	8	6	2	0,25	25
Canada	6	3	3	0,5	50
Denmark	6	4	2	0,33333333	33
New Zealand	6	3	3	0,5	50
Poland	6	6	0	0	0
France	5	5	0	0	0
Italy	5	3	2	0,4	40
Norway	5	4	1	0,2	20
Spain	5	5	0	0	0
Greece	4	3	1	0,25	25
Brazil	3	1	2	0,66666667	67

### 3.3 The most frequently cited countries

According to Aksnes, Langfeldt, and Wouters, citations are increasingly employed as performance indicators in research policy and the academic system. They are typically viewed as a measure of a study's impact or quality (21).

**Figure 3:**
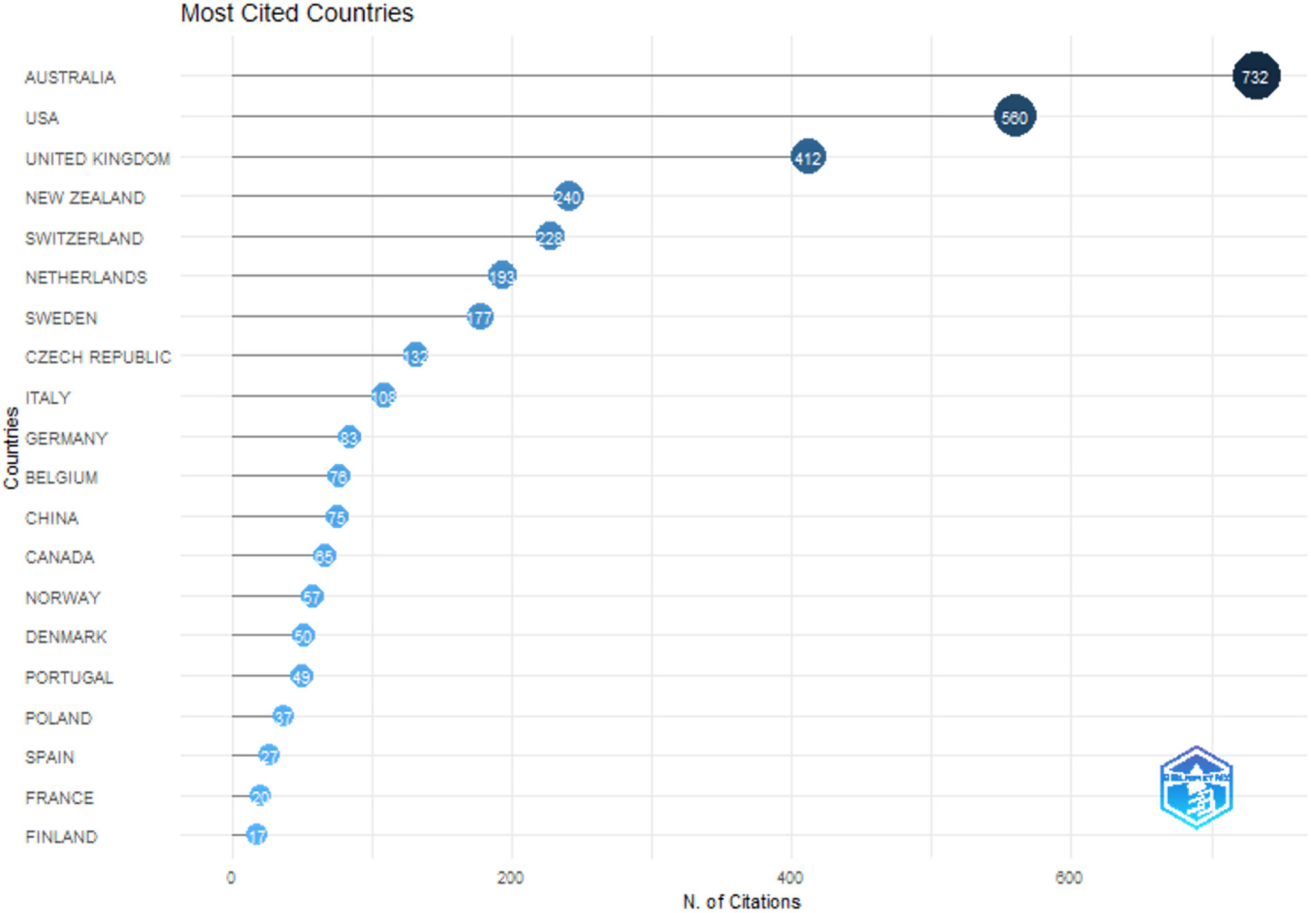
Most cited countries.

The analysis of total citations (TC) and average article citations (22) provides valuable insights into the research output and impact of different countries in the field of lex sportiva and sports governance. Total citations represent the cumulative impact of all articles from a specific country, while average article citations measure the influence of individual publications. These metrics highlight significant patterns and trends in global research contributions (Figure 3 and Table 4).

**Table 4:** Most cited countries.

Country	TC	Average article citations
Australia	732	23,6
USA	560	20,7
United Kingdom	412	12,5
New Zealand	240	40
Switzerland	228	25,3
Netherlands	193	21,4
Sweden	177	19,7
Czech Republic	132	66
Italy	108	21,6
Germany	83	5,2
Belgium	76	9,5
China	75	4,4
Canada	65	10,8
Norway	57	11,4
Denmark	50	8,3
Portugal	49	16,3
Poland	37	6,2
Spain	27	5,4
France	20	4
Finland	17	5,7

Australia leads with the highest total citations (732), followed by the USA (560) and the United Kingdom (412), indicating these countries’ substantial contributions to impactful research. However, the Czech Republic, despite having fewer total citations (132), stands out with an impressive average citation score of 66, suggesting high-quality and influential publications. On the other hand, countries such as Finland (17), France (20), and Spain (27) demonstrate limited overall impact, either due to fewer publications or lower engagement in this research domain.

New Zealand (40) and Switzerland (25.3) also exhibit high average article citations, emphasizing the substantial influence of their research outputs despite smaller publication volumes. Meanwhile, Australia (23.6) and the USA (20.7) maintain a strong balance between high-volume and high-quality research contributions. In contrast, Germany (5.2), China (4.4), and France (4) reflect lower average citation rates, which could suggest either lower research impact or the presence of fields with limited citation opportunities.

Several patterns emerge when examining the data. High-output and high-impact leaders like Australia, the USA, and the United Kingdom display robust research ecosystems focused on global relevance and influence. In contrast, countries such as the Czech Republic, New Zealand, and Switzerland represent high-impact but low-output contributors, producing niche research with exceptional quality and recognition. Large contributors with moderate impact, such as China, highlight the complexities of producing high-volume research that may take time to gain recognition or pertain to less-cited fields. On the other hand, countries like France, Spain, and Poland, with both low output and low impact, underscore the need for more engagement and investment in these areas.

Notable outliers include the Czech Republic, with an exceptional average citation score of 66, likely driven by landmark studies or publications in highly cited areas. Similarly, New Zealand and Switzerland demonstrate strong average citation rates, reflecting their research's global influence and high quality, despite lower total output.

These trends illustrate how research contributions and their impact can vary significantly across countries, shaped by factors such as the focus of research, the quality of studies, and the dynamics of global academic discourse. Understanding these patterns is essential for fostering collaboration and directing future research efforts in the field of lex sportiva and sports governance.

### 3.4 Keyword co-occurance

**Figure 4:**
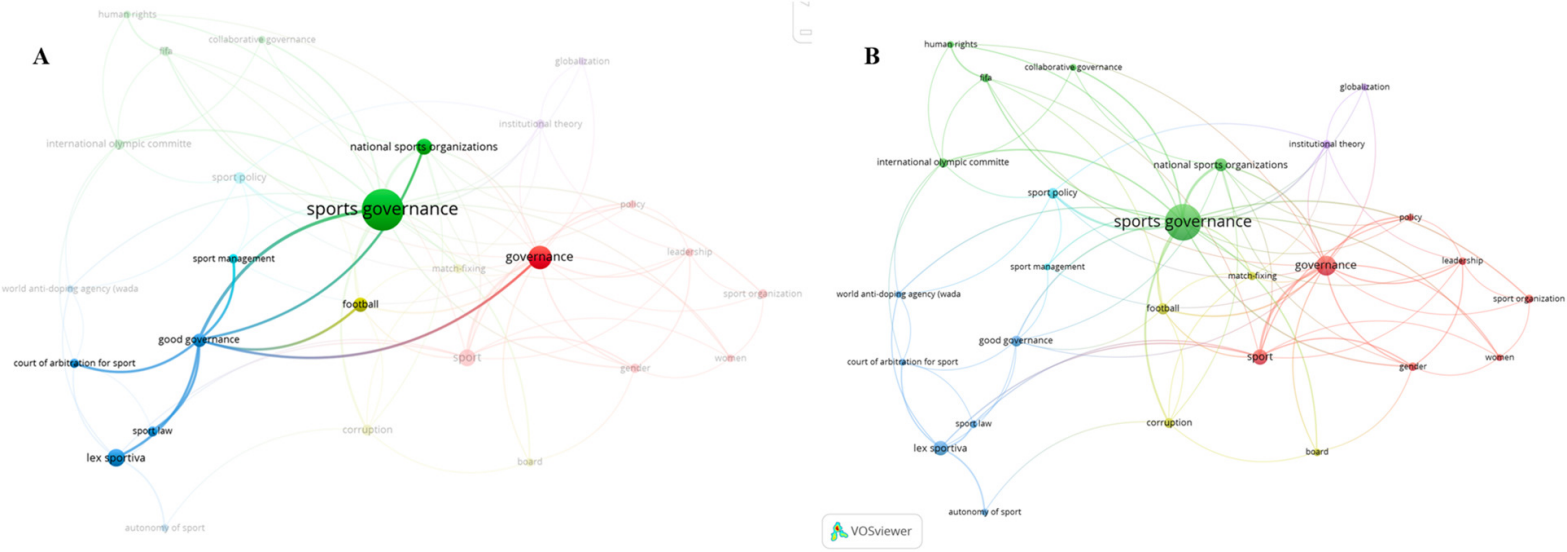
Author keyword co-occurence **(A)** good governance as the intersetcion of sports governance and lex sportiva and **(B)** overall co-occurence of author keywords.

The VOSviewer visualization of author keyword co-occurrence, as shown in Figure 4, highlights the intricate connections between sports governance and lex sportiva, with “good governance” emerging as the primary conceptual bridge that links the organizational focus of the former with the legal frameworks of the latter. The analysis reveals a total of six clusters, each representing distinct but interrelated themes within the broader research landscape.

To create this visualization, several key steps were followed. First, a map was generated based on bibliographic data retrieved from the Scopus database. The type of analysis conducted was co-occurrence, with authors’ keywords as the unit of analysis and the counting method set to full counting. A VOSviewer thesaurus file was uploaded to ensure consistent terminology, and a minimum threshold of five occurrences per keyword was applied. Out of 874 identified keywords, 27 met this threshold and were selected for inclusion in the analysis. These steps ensured the generation of a focused and meaningful visualization that captures the core themes of the dataset.

Within the field of sports governance, good governance according to “Good governance principles for UEFA member associations” (23) emphasizes 10 principles such as: Clear strategy; Statutes; Stakeholder involvement; Promotion of ethical values, integrity and good governance; Professionalism of committee structures; Administration; Accountability; Transparency in financial matters and corporate documents; Compliance; Volunteer programmes. Good governance has become a key focus in contemporary sports policy and academic research, underscoring the importance of transparency, accountability, and ethical leadership in sports organizations. Recent scholarship has developed conceptual models and offered empirical insights that inform the evolving understanding of what constitutes “good governance” in sport. Notable contributions have been made by scholars such as Arnout Geeraert, Frank van Eekeren, Ashley Thompson, Jean-Loup Chappelet, and Maja Mrkonjic. Their work will be discussed in greater detail in the following chapter.

In contrast, within lex sportiva, the term reflects adherence to legal standards, fairness in arbitration, and the consistent application of rules by international sports bodies like the International Olympic Committee (IOC) and FIFA. This shared emphasis on governance standards highlights the overlapping concerns of these domains, particularly in areas where regulatory compliance, ethical practices, and legal principles intersect. Studies addressing governance reforms, regulatory oversight, and ethical challenges in sports often draw from both disciplines, exploring how governance frameworks and legal principles shape the management and regulation of sports bodies.

The presence of six distinct clusters in the visualization further illustrates the interdisciplinary nature of this research. These clusters represent thematic groupings where specific aspects of sports governance and lex sportiva are studied, with overlapping nodes demonstrating their interconnectedness. For example, topics such as international arbitration, anti-corruption measures, and compliance with governance standards frequently appear as shared concerns, exemplifying the convergence of these fields.

This connection highlights emerging opportunities for research, particularly in examining how principles of good governance are implemented within the legal frameworks of lex sportiva. Areas such as financial transparency, athlete representation, and the governance of international sports events offer fertile ground for exploring the mutual influence of legal standards and governance mechanisms. Moreover, the increasing attention to good governance within sports governance and lex sportiva reflects the growing recognition of their critical role in addressing global challenges in sports management and regulation.

### 3.5 Collaborative patterns through citation analysis of key contributors

**Figure 5:**
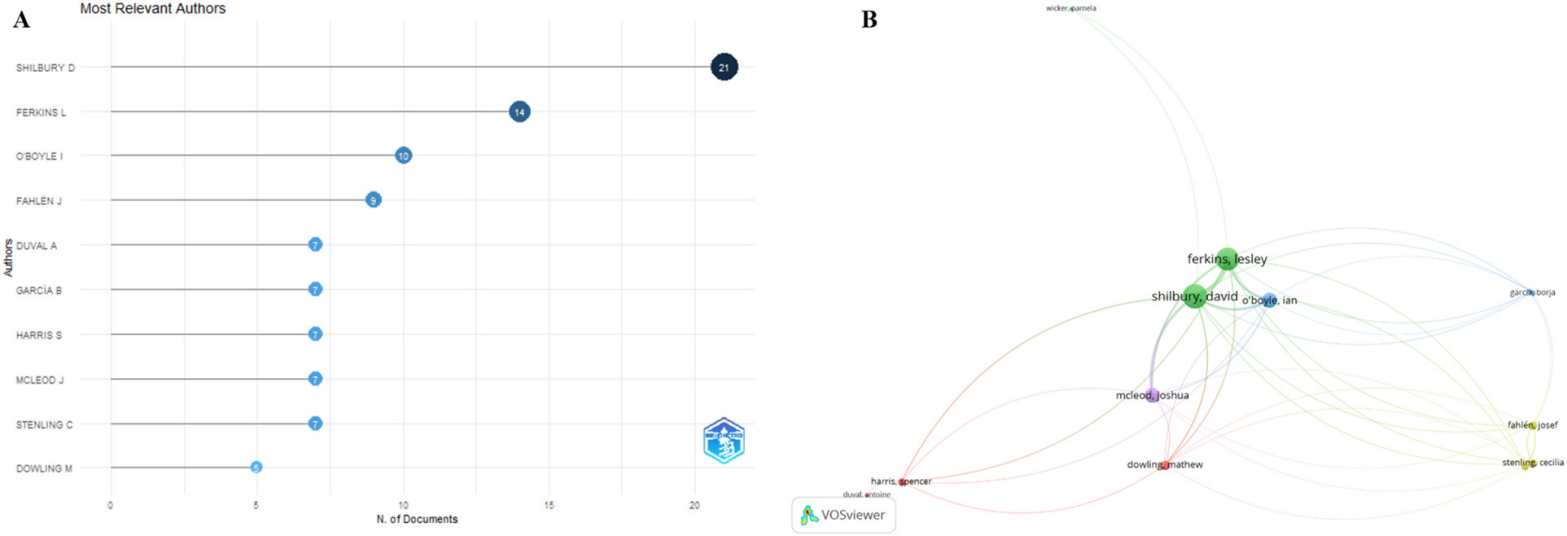
Citation analysis of influential contributors **(A)** most relevant authors and **(B)** most relevany authors by clusters.

 Figure 5 represents a citation analysis of authors, highlighting influential contributors in the research fields of lex sportiva and sports governance.

To generate the map, a citation analysis was conducted with the unit of analysis set to authors. A VOSviewer thesaurus file was uploaded to standardize author names, addressing inconsistencies such as variations in spellings or initials. Specific thresholds were applied to ensure the inclusion of authors with significant contributions: each selected author needed to have a minimum of five publications and at least five citations. These criteria filtered the dataset to 13 authors who demonstrated substantial academic influence.

Among the most influential authors in the dataset, the ten leading contributors are Shilbury (21 articles), Ferkins (14 articles), O'Boyle (10 articles), Fahlen (9 articles), and Duval (7 articles), Garcia (7 articles), Harris (7 articles), McLeod (7 articles), Stenling (7 articles), Dowling (5 articles).Their high publication output, coupled with significant citation impact, positions them as key figures shaping the academic discourse in lex sportiva and sports governance.

The resulting citation map depicts relationships between authors based on the frequency with which their work is cited. Authors with higher citation counts and stronger connections appear centrally in the visualization, indicating their pivotal role in shaping the research landscape. Dense clusters within the map suggest thematic alignment or frequent citation among authors, reflecting collaborative research communities or shared areas of focus. Conversely, authors positioned on the periphery may represent niche contributors or researchers with limited connections to the core network.

This visualization provides valuable insights into the intellectual structure of the research fields, identifying key contributors and the patterns of influence among them. It highlights the collaborative dynamics and thematic overlaps that define the academic discourse on lex sportiva and sports governance. Researchers can use this analysis to identify foundational works, explore emerging trends, and establish connections with thought leaders in these domains as the map offers a comprehensive understanding of the scholarly landscape in this area of study.

### 3.6 Most relevant sources

**Figure 6:**
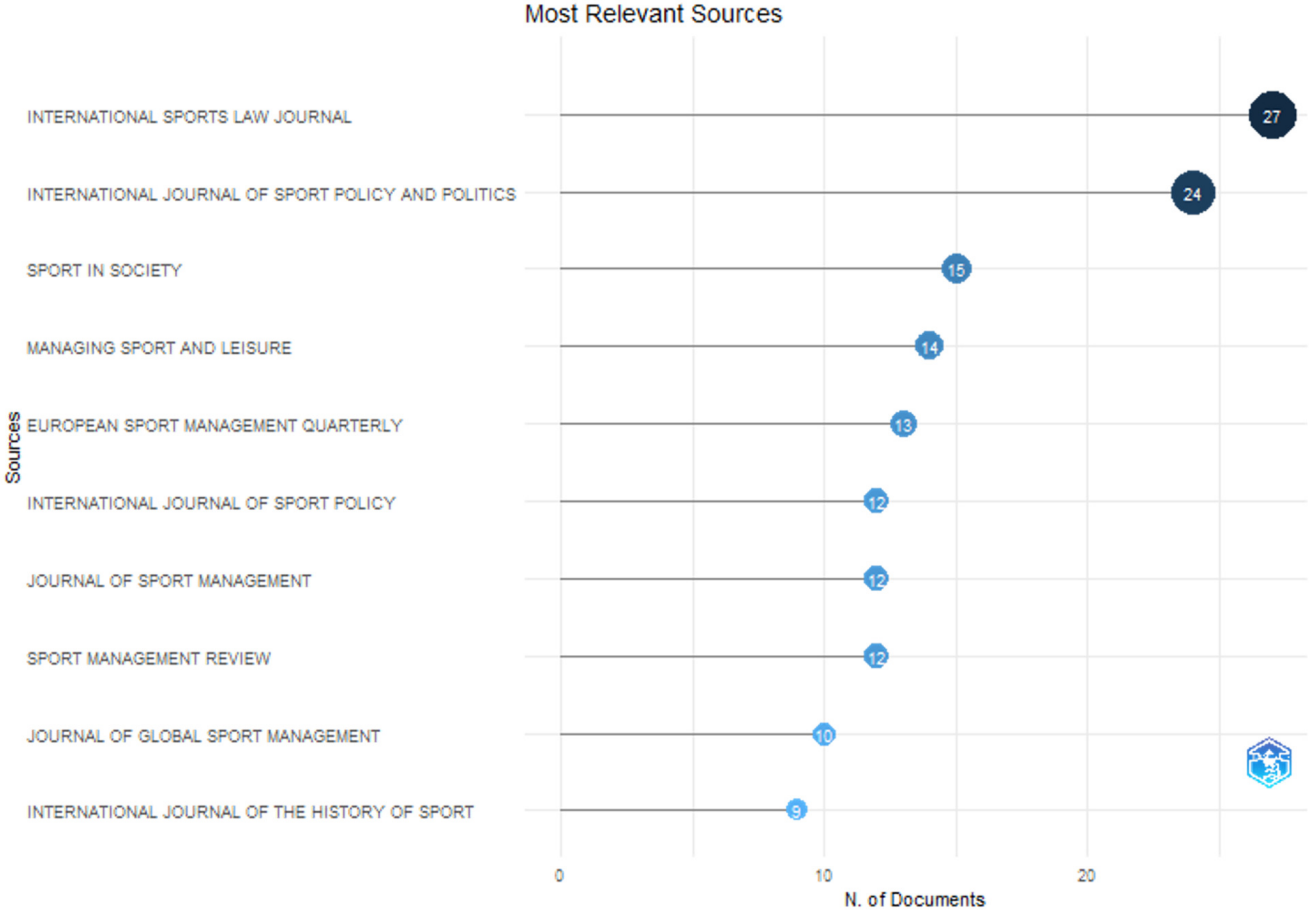
Key journals publishing research in sports law and sports governance.

An essential aspect of this bibliometric study is the identification of key journals publishing research in sports law and sports governance (Figure 6). The most influential source is the International Sports Law Journal, which has the highest number of articles (27), emphasizing its dominance in the field. It is followed by the International Journal of Sport Policy and Politics (24), Sport in Society (15), Managing Sport and Leisure (14), and European Sport Management Quarterly (13). These journals serve as primary platforms for disseminating research on legal and governance aspects of sports.

Journals also have their own H-Index scores. Publishing in a high H-index journal maximizes the chances of being cited by other authors and, consequently, may improve an individual researcher's personal H-index score. Knowing the H-index score of journals of interest is useful when searching for the right one to publish a paper (24). Therefore, to provide further context, data from the Scimago Journal & Country Rank (25) were examined to assess the H-index of these journals based on the latest available data.

The International Sports Law Journal has an H-index of 13, indicating moderate impact within the academic community. The International Journal of Sport Policy and Politics has an H-index of 40, showing a broader influence, while Sport in Society has an H-index of 50, reflecting its strong academic recognition. The Managing Sport and Leisure journal has an H-index of 43, whereas the European Sport Management Quarterly boasts an H-index of 49, making it one of the most influential sources in this field. These metrics underscore the significance of these journals in shaping scholarly discourse and advancing research on sports governance and lex sportiva.

These findings underscore the concentration of research dissemination within a select group of journals, which play a crucial role in shaping academic discourse in sports law and governance. Future studies may consider diversifying publication venues to enhance the global reach and interdisciplinary engagement of research in this area.

## 4 Discussion

The findings of this bibliometric analysis provide a comprehensive overview of the research landscape in sports law and governance, highlighting key trends, influential contributors, and thematic intersections. The increasing volume of publications over recent decades indicates a growing academic interest in these fields, particularly in response to global sports commercialization, legal disputes, and governance challenges. While traditionally treated as separate domains, sports law and governance are increasingly converging—particularly through the lens of “good governance”, as underscored by the *keyword co-occurrence analysis*. This intersection is especially evident in studies examining regulatory frameworks, compliance mechanisms, and ethical considerations in sports administration.

Good governance in sports is widely recognized as essential for maintaining public trust and the integrity of sports organizations. In 2017, European Commissioner Tibor Navracsics emphasized the critical need for transparency, accountability, and stakeholder involvement, particularly in light of governance failures—most notably in football—that have undermined the reputation of sport (26). UEFA, as the umbrella organization for European football, actively promotes a system of checks and balances to prevent excessive concentration of power and to ensure continuity in decision-making. Its Good Governance Principles for UEFA Member Associations are specifically designed to support governance reforms at the national level (27).

Mislav Mataija highlights several cornerstones of sports governance development in Europe, notably the 2022 “Arrangement for Cooperation” between the European Commission and UEFA. This agreement reinforces the link between good governance and the autonomy of sports organizations, promoting solidarity by reinvesting professional competition revenues into grassroots development and aligning with EU priorities such as climate action, equality, and social inclusion. UEFA's initiatives, including support for the EU Green Deal, illustrate how football can be leveraged to drive positive social change. Similarly, the European Commission, Council of Europe, and UNESCO have all championed governance reforms, with some countries now requiring sports bodies to adopt governance codes as a condition for receiving public funding (27).

A fundamental aspect of good governance is the establishment of effective judicial and dispute resolution mechanisms within sports organizations. This aligns with Juan Antonio Samaranch's vision of resolving sporting disputes within the sporting community (28). However, in some countries, such as Latvia, such systems remain underdeveloped. Despite challenges including fragmented governance structures, inconsistent funding, and inadequate infrastructure, there is optimism for reform, driven by EU policies and the efforts of international organizations such as UEFA and the IOC. Strengthening governance structures and establishing stable, long-term funding models are essential steps toward ensuring ethical, transparent, and accountable management in sports.

The concept of good governance has been the subject of extensive scholarly analysis. Geeraert and van Eekeren (29) argue that understanding good governance requires both theoretical reflection and practical assessment, emphasizing the importance of considering diverse definitions and justifications—both moral and instrumental—and recognizing the dilemmas and trade-offs inherent in translating governance principles into practice. Their work advocates for a systemic and reflexive approach to governance reform in sports organizations. Complementing this, Chappelet and Mrkonjic (30) provide a comprehensive synthesis of existing governance frameworks, surveying how international and national bodies, NGOs, and academic literature define and operationalize governance in sport. Their review identifies both commonalities and divergences in principle interpretation, highlighting the evolving nature of the governance discourse.

Further reinforcing these perspectives, Thompson et al. (31) conducted a systematic review of governance principles in sport, categorizing core elements such as transparency, accountability, participation, integrity, and effectiveness. Their findings underscore the contextual variability in governance implementation and call for tailored frameworks that balance universal values with specific organizational and cultural realities. Collectively, these sources deepen the understanding of good governance as not only a normative ideal but also a contested and dynamic field of practice, central to both the regulatory and operational dimensions of modern sport.

As outlined in the works mentioned above, good governance in sport refers to the establishment of transparent, accountable, and ethically grounded systems and processes within sports organizations. It aims to ensure integrity, fairness, and effective operations by aligning organizational practices with legal standards, while fostering inclusivity and building stakeholder trust.

Governance reforms, dispute resolution mechanisms, and regulatory oversight illustrate the extent to which legal principles influence governance structures within sports organizations. These findings align with broader discussions on the European Sport Model, which promotes transparency, accountability, and stakeholder involvement across professional and grassroots levels. These organizational developments naturally lead into a broader examination of how European Union law shapes and legitimizes sports governance structures across the continent.

The development of sports law within the European Union offers a compelling illustration of the interplay between traditional market-regulating legal frameworks and the internally developed rules of private sporting bodies that shape global sports governance. Rather than positioning itself in opposition to sport's ‘internal law’, EU law grants it a form of conditional autonomy. In defining the parameters of this autonomy, EU institutions—most notably the Court of Justice and the European Commission—have had to construct a notion of legitimate sports governance, despite the lack of explicit Treaty provisions directly addressing this domain (32).

The Advocate General's Opinion in Case C-333/21 European Superleague Company underscores the significance of Article 165 TFEU, which enshrines a ‘constitutional’ recognition of the European Sports Model. This model encompasses several foundational principles shared across many sporting disciplines in Europe, including football (33). As García observes, the EU generally adopts a cautious approach to engaging with the social dimensions of sport, opting instead to focus on overseeing and subtly shaping governance frameworks to ensure their compatibility with EU legal standards (34).

The *citation analysis* further reinforces the interdisciplinary nature of research in claimed fields. The identification of key scholars contributing significantly to both domains suggests a cross-pollination of ideas between legal and governance frameworks. The presence of multiple research clusters indicates diverse focal points, ranging from international arbitration and competition law to ethical governance and institutional oversight. This thematic diversity highlights the increasing complexity of sports regulation and the need for integrated approaches that address both legal and managerial dimensions.

Moreover, the *analysis of publication trends* suggests that high-profile legal cases and governance failures have played a crucial role in shaping research agendas. The acceleration in publication volume post-2010 coincides with major sporting scandals, regulatory shifts, and the increasing role of supranational bodies like the Court of Arbitration for Sport (CAS) and the European Union in sports regulation. These developments have spurred academic discourse on the effectiveness of existing legal and governance frameworks, particularly in ensuring athlete rights, financial transparency, and anti-corruption measures.

The *country-wise analysis of citations and collaborations* underscores the dominance of Australia, the USA, and the UK as leading contributors to sports law and governance research. These countries not only produce high volumes of publications but also demonstrate strong international collaboration networks, reinforcing their role in shaping global discussions. Interestingly, countries such as the Czech Republic and Switzerland, despite lower overall output, exhibit high average citations per publication, indicating a focus on high-impact research. The varying levels of international collaboration among different nations highlight the uneven distribution of expertise and research efforts, with some countries engaging more actively in global discussions while others remain largely focused on domestic concerns. In contrast to disciplines such as political science or management studies that, as authors suggest, usually demonstrate a more globally distributed pattern of co-authorship and citation, the field of sports law and governance remains relatively concentrated in a few Anglophone countries. This concentration suggests a degree of disciplinary insularity, shaped by national legal frameworks, language barriers, and the localized nature of legal scholarship. Contributing factors may include disparities in research funding, differences in publication practices, and the uneven prominence of sports law and governance in national policy agendas. As also mentioned by Glänzel and Schubert, scientific collaboration is clearly stimulated (or hindered) by national, regional and global political interests (35). Moreover, the dominance of English-language journals indexed in Scopus may limit the visibility and citation impact of scholars from non-English-speaking countries. These findings underscore the importance of fostering greater international collaboration and promoting the inclusion of diverse perspectives to enrich the global discourse in this field.

*Keyword analysis* further highlights the thematic progression of research in the field of sports governance. Core concepts such as “sports law,” “sports governance,” and “good governance” remain foundational. At the same time, emerging themes—such as gender diversity in governance, specifically the representation of women on the boards of sport governing bodies (36); match-fixing, defined as attempts to improperly influence the outcome or course of a sporting event for undue advantage (37); and financial fair play, a UEFA regulation designed to ensure financial transparency, timely payments, and adherence to break-even requirements for long-term club sustainability (38)—reflect the evolving challenges faced by the sports industry. The dominance of governance-related keywords reinforces the idea that legal structures and regulatory mechanisms are becoming increasingly central to discussions on sports governance.

Moreover, the VOSviewer visualizations reveal six major clusters within the research field, each representing a different aspect of sports law and governance. The strongest linkages occur between governance-related keywords, further emphasizing the fundamental role that legal considerations play in structuring sports organizations and ensuring compliance with international standards.

While the bibliometric results provide valuable insights into the evolution of research in sports law and governance, certain limitations must be acknowledged. The reliance on Scopus as the primary data source, while comprehensive, may exclude relevant contributions indexed in other databases. Additionally, the study's focus on keyword co-occurrence and citation analysis, though informative, may not capture the full depth of qualitative insights embedded within individual studies. Future research could benefit from expanding the dataset to include multidisciplinary perspectives and employing qualitative content analysis to complement the quantitative findings.

In conclusion, this study highlights the growing importance of integrating legal and governance perspectives in sports research. The emergence of “good governance” as a unifying theme underscores the need for continued exploration of how legal principles shape governance structures in sports organizations. As the field continues to evolve, future studies should focus on emerging challenges such as digital transformation in sports governance, the role of artificial intelligence in regulatory compliance, and the implications of geopolitical shifts on international sports law. By advancing interdisciplinary research, scholars and practitioners can contribute to the development of more effective and ethically sound governance models in the global sports industry.

## 5 Conclusion

This study offers a comprehensive bibliometric analysis of lex sportiva and sports governance research, highlighting key trends, influential contributors, and emerging themes. The findings underscore the following:

1. The evolution of research in sports law and governance reflects a growing academic interest, particularly in response to globalization, legal controversies, and governance challenges in sports. The significant increase in publication volume since 2010 aligns with major developments in international sports, such as regulatory changes, commercialization, and governance failures as well as high-profile scandals.
2. The intersection between sports governance and lex sportiva remains underexplored, with “good governance” serving as the primary conceptual link between these fields. The keyword analysis reveals that studies addressing governance reforms, legal compliance, and ethical oversight frequently integrate elements from both domains, suggesting that future research should further examine their interplay.
3. Geographic disparities exist in research contributions, with Australia, the USA, and the UK emerging as the dominant players in sports law and governance scholarship. These countries not only produce the highest volume of publications but also engage in extensive international collaborations, reinforcing their role in shaping global academic discourse.
4. Smaller research economies, such as the Czech Republic, New Zealand, and Switzerland, demonstrate high research impact despite lower publication output. The high average citation rates in these countries indicate the presence of landmark studies that significantly contribute to the field.
5. The presence of six thematic research clusters highlights the multidisciplinary nature of sports law and governance studies. These clusters represent diverse areas of inquiry, including legal arbitration, compliance mechanisms, and governance ethics, demonstrating the broad scope of the field and its relevance to multiple disciplines.
6. The identification of key journals, such as the International Sports Law Journal and the European Sport Management Quarterly, underscores the concentration of research dissemination within a select group of sources. While these journals play a crucial role in shaping academic discourse, expanding publication venues could enhance the global reach and interdisciplinary engagement of sports law and governance research.
7. Future research should focus on emerging governance and legal challenges in sports, e.g., gender diversity in governance, financial regulations, digital transformation, the use of artificial inteligence, sports diplomacy. As sports organizations increasingly adopt new technologies and regulatory frameworks, continued interdisciplinary research will be essential to addressing evolving governance complexities.
8. The limited number of search results when combining “sport governance” with “lex sportiva” or “sport law” indicates a research gap in the direct integration of these fields. The absence of results for “sport governance” AND “lex sportiva” and the retrieval of only five articles for “sport governance” AND “sports law” suggest that these domains are often studied separately rather than as interconnected disciplines. This highlights an opportunity for future research to explicitly examine the legal dimensions of sports governance and the governance implications of lex sportiva, fostering a more comprehensive understanding of their relationship.

As the sports industry continues to face governance and legal challenges, the role of interdisciplinary research in shaping policy and regulatory frameworks will become even more critical. This study serves as a foundational resource for scholars, policymakers, and practitioners seeking to navigate the complexities of sports law and governance in a rapidly evolving global landscape.

## Data Availability

The original contributions presented in the study are included in the article/Supplementary Material, further inquiries can be directed to the corresponding author.
